# Bone histomorphometric and immunohistological analysis for hyperostosis in a patient with SAPHO syndrome: A case report

**DOI:** 10.1016/j.bonr.2020.100296

**Published:** 2020-07-15

**Authors:** Shun Watanabe, Naoki Sawa, Hiroki Mizuno, Rikako Hiramatsu, Noriko Hayami, Masayuki Yamanouchi, Tatsuya Suwabe, Junichi Hoshino, Takeshi Fujii, Toshihide Hirai, Tomoka Hasegawa, Norio Amizuka, Yoshifumi Ubara

**Affiliations:** a Nephrology Center, Toranomon Hospital, Kanagawa, Japan; b Okinaka Memorial Institute for Medical Research, Toranomon Hospital, Tokyo, Japan; c Department of Pathology, Toranomon Hospital, Tokyo, Japan; d Department of Orthopaedic Surgery and Spinal Surgery, Graduate School of Medicine, the University of Tokyo, Tokyo, Japan; e Hard Tissue Developmental Biology Department Graduate School of Dental Medicine and Faculty of Dental Medicine, Hokkaido University, Sapporo, Japan

**Keywords:** SAPHO, synovitis-acne-pustulosis-hyperostosis-osteitis, ALP, alkaline phosphatase, TRAP-5B, tartrate-resistant acid phosphatase 5B, MMP-9, matrix metallopeptidase 9, CT, computed tomography, RF, rheumatoid factor, CCP, cyclic citrullinated peptide, ANA, antinuclear antibody, C3, complement component 3, C4, complement component 4, CH50, total complement, IgG, immunoglobulin G, IgA, immunoglobulin A, IgM, immunoglobulin M, SCCH, sternocostoclavicular hyperostosis, PPP, palmoplantar pustulosis, SAPHO syndrome, Hyperostosis, ALP, Almoplantar pustulosis, Osteoblasts

## Abstract

A 56-year-old Japanese woman with a history of palmoplantar pustulosis was admitted for examination due to left femur pain. Radiography and computed tomography showed thickening of the bone on the outer portion of the left femur. Bone scintigraphy of the left femur showed intense radioactive uptake. Consequently, the patient was diagnosed with SAPHO syndrome. Bone histomorphometric analysis of the left femur showed cancellous bone with thickened cortical bone. Whilst normal bone shows cancellous bone with double labeling (normal turn over), and cortical bone with no labeling (low turn over, adynamic state), this case presented with both cancellous and cortical bone with marked double labeling (indicating high turn over), abundant osteoid and woven bone. Immunohistological analysis showed that cells lining the bone surface consisted of osteoblasts and were positive for alkaline phosphatase (ALP). Few to little of these cells were positive for tartrate-resistant acid phosphatase (TRAP)-5B, cathepsin K and matrix metallopeptidase 9 (MMP-9). These results indicate that, in this case study, excessive production of osteoblasts contributed to hyperostosis of the left femur, with abundant osteoid and woven bone. This type of bone formation in SAPHO syndrome is not lamellar bone seen in normal bone, but rather fragile and mechanically weak bone, resulting in bone pain. Doxycycline may be a therapeutic option for bone pain in this patient.

## Highlights

• The patient with thickened left femur was diagnosed with SAPHO syndrome.
• Bone histomorphometric analysis showed marked bone formation.
• Immunohistological analysis showed excessive production of osteoblasts.
• This type of bone formation makes fragile and mechanically weak bone.

## 1 Introduction

Synovitis-acne-pustulosis-hyperostosis-osteitis (SAPHO) syndrome is a rare inflammatory disorder of the bone, joints, and skin, first described as a syndrome in 1987 (Rukavina, 2015). The disease is believed to be a disease of a combination of autoimmune diseases, environmental factors and genetic factors, however the pathogenesis of SAPHO syndrome remains unclear (Cianci et al., 2017). Hyperostosis is one of main clinical features of SAPHO syndrome, however pathology findings of hyperostosis are rarely reported. In the present study, the bone histology of hyperostosis in a patient with SAPHO syndrome was analyzed using histomorphometrical and immunohistological methods.

## 2 Case report

In March 2017, a 56-year-old Japanese woman was diagnosed by a dermatologist in the outpatient clinic of the neighboring hospital with palmoplantar pustulosis (PPP) including skin ulcers and erosion on both feet and fingers. Following this diagnosis, the patient began to suffer left leg pain, with the pain increasing. Prednisolone was administrated at the dose of 20 mg daily, but the pain continued. This resulted in the patient being admitted to the authors' hospital for the examination of the left femur pain in January 2018.

On admission, the patient was 156.2 cm tall and weighed 55.4 kg, with a blood pressure of 124/65 mmHg. Laboratory tests were conducted including serum creatinine (1.19 mg/dL), and C-reactive protein (0.2 mg/dL). Rheumatoid factor (RF) was negative at 1 U/mL (normal: <10 U/mL), cyclic citrullinated peptide (CCP) antibodies were negative at <0.5 U/mL (normal <4.5 U/mL), and all autoantibodies were negative, including antinuclear antibody (ANA). Serum complement component 3 (C3) was 106 mg/dL (normal: >86 mg/dL), complement component 4 (C4) was 18 mg/dL (normal: >17 mg/dL), and total complement (CH50) was 44 U/mL (normal: >30 U/mL). The serum level of immunoglobulin G (IgG) was 1705 mg/dL (normal: 870 to 1700 mg/dL), immunoglobulin A (IgA) was 265.9 mg/dL (normal: 110 to 410), and immunoglobulin M (IgM) was 61.2 mg/dL (normal: 33 to 190). Alkaline phosphatase (ALP) was 258 IU/L (normal: 117 to 350), bone ALP was 60.3 μg/L (normal: 3.8 to 22.6), tartrate-resistant acid phosphatase (TRAP)-5B was 848 mU/dL (normal: 250 to 760), and intact parathyroid hormone was 117 pg/mL (normal: 25 to 117) (Table 1).

**Table 1:** Laboratory finding on admission. AST, asparate aminotransferase; ALT, alanine transaminase; LDH, lactate dehydrogenase; ALP, alkaline phosphatase; GTP, glutamyl transferase; eGFR, estimated glomerular filtration ratio; HDL, high density lipoprotein; LDL, low density lipoprotein; CRP, C-reactive protein. iPTH, intact parathyroid hormone.

Normal range			
Age	56		
Body weight	54.5		kg
White blood cells	7700	3200–7900	/μL
Hemoglobin	9.8	11.3–15.0	g/dL
Hematocrit	30.8	34.0–46.3	%
Platelets	223	155–350	*10^3^/μL
Total protein	6.3	6.9–8.4	g/dL
Albumin	3.2	3.9–5.2	g/dL
AST	21	13–33	IU/L
ALT	22	7–23	IU/L
LDH	119	119–229	IU/L
ALP	258	117–350	IU/L
γ-GTP	28	9–109	IU/L
Urea nitrogen	21	8–21	mg/dL
Creatinine	0.33	0.46–0.78	mg/dl
eGFR	151.9	>90	ml/min/1.73m^2^
Uremic acid	5.7	2.5–7.0	mg/dL
Triglyceride	35	30–150	mg/dL
Total cholesterol	96	122–240	mg/dL
HDL cholesterol	46	35–70	mg/dL
LDL cholesterol	34	<140	mg/dL
Na	143	139–146	mmol/L
K	3.7	3.7–4.8	mmol/L
Cl	109	101–109	mmol/L
Corrected Ca	10.2	8.7–10.1	mg/dL
P	4.6	2.8–4.6	mg/dL
CRP	0.2	<0.14	mg/dL
Ferritin	101	5–80	μg/L
RF	1	<10	U/mL
Anti-CCP antibody	<0.5	<4.5	U/mL
ANA	<40	<40	
C3	106	>8.6	mg/dL
C4	18	>17	mg/dL
CH50	44	>30	U/mL
IgG	1705	870–1700	mg/dL
IgA	265.9	110–410	mg/dL
IgM	61.2	33–190	mg/dL
Bone specific ALP	90.8	3.8–22.6	μg/L
TRACP-5b	965	250–760	mU/dL
1,25dihydroxyvitaminD	54	20.0–60.0	ng/L
Corrected Ca	9	8.7–10.1	mg/dL
iPTH	177	15–65	pg/mL
Osteocalcin	26.7	14.2–54.8	ng/mL
Deoxypyridinoline(Urine)	12.2	2.8–7.6	nmol/mmol･Cr

**Fig. 1:**
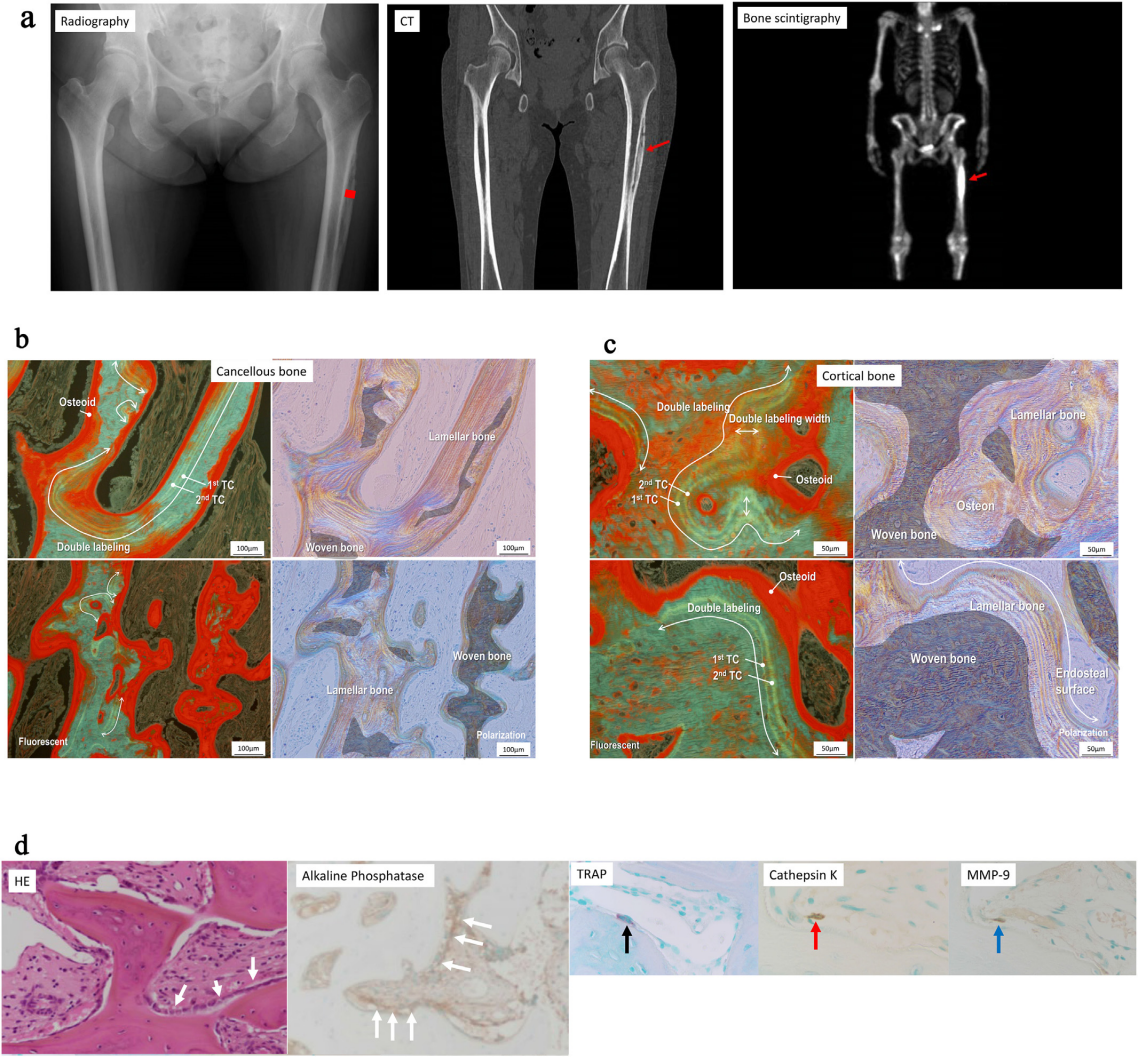
**a** Plain radiography and computed tomography. Bone thickening (arrow) on the outer portion of the left femur. Biopsy area(square). Bone scintigraphy with 99mTc–methylene diphosphonate. Intense uptake (arrow). **b** Bone biopsy (fluorescent and polarization) in the cancellous bone at two sites indicating rapid bone formation. Tetracycline double labeling (white line) along lamellar bone surfaces. Red area indicates osteoids. Woven bone is massive. **c** Bone biopsy (fluorescent and polarization) in the cortical bone at two sites. Tetracycline double labeling along lamellar bone surfaces with increased osteoid layer (red area) covered by osteoblast-like mononuclear cells. Woven bone is massive. **d** Hematoxylin and eosin staining staining showing cell lines (white arrow) covering the bone surface, including osteoblasts that stained positive for ALP, and osteoclasts that stained positive for TRAP-5B (black arrow), cathepsin K (red arrow) and MMP-9 (blue arrow).

Plain radiography and computed tomography showed thickening of the bone on the outer portion of left femur, which bone scintigraphy with 99mTc–methylene diphosphonate showed intense radioactive uptake (Fig 1a).

A diagnosis of SAPHO syndrome was given due to characteristic skin manifestations (including palmoplantar pustulosis) and bone manifestations (including hyperostosis). Bone biopsy was performed to investigate the pathogenesis of this disease after tetracycline double labeling with the schedule of 02-7-02-25.

Bone biopsy was performed as follows. A 5 cm vertical skin incision was made just above the lesion. Incision of the vastus lateralis and periosteum revealed incorrect bone cortex. The part was perforated to 1.5 cm × 0.8 cm. Upon reaching the defect in the bone cortex, there were a hematoma and a tissue looked like normal bone inside, from which a specimen was taken. No abscess or infected granulation tissue were detected.

Bone histomorphometrical analysis and immunohistological analysis was performed according to previously described methods (Hiramatsu et al., 2013). The normal value of bone histomorphometrical analysis is referred according to the report of Recker et al. (1988). We must note the followings: It is not the value of the femoral diaphysis, but the value of iliac bone. In addition to it has not been age-adjusted.

## 3 Bone histomorphometrical and immunohistological analysis

The femur was biopsied including cancellous bone with a thickened cortical bone, and mineralized bone including large amounts of woven bone (Fig. 1b and c). In the cancellous bone, tetracycline double labeling was observed in the majority of the lamellar bone surfaces. Bone mineral apposition rate [(double labeling width/number of days of first labeling holiday] was increased by 2.28 μm/day. (normal, <1 μm/day) and osteoid volume [(osteoid volume/bone volume) × 100] was increased by 23.1% (normal, 3.1 ± 2.0%). The osteoid surface [(osteoid surface/bone surface) × 100] was also increased by 98.7%(normal, 16.7 ± 7.0%)as to was osteoid thickness by 19.3 μm (normal, 9.16 ± 2.0) (Fig. 1b). The ratio of total bone volume to total tissue volume was increased by 44.1% (normal, 20.8 ± 1.5%). The ratio of fibrous tissue volume to total volume was increased by 13.4% (normal, <0.5%). The ratio of eroded surface to bone surface, however, was decreased (1.1%; normal, 5.6 ± 1.9%). The number of osteoblast-like mononuclear cells was increased, however osteoclast-like multinucleated giant cells were not visible near the inside of the bone surface. Bone formation rate/trabecular bone surface was markedly increased (0.384; normal,0.015 ± 0.008)(Fig. 1b). The ratio of woven bone to bone volume was markedly increased (43.8%; normal, 0%).

In cortical bone, tetracycline double labeling was observed along the lamellar bone surfaces, with increased osteoid layer, covered by numerous osteoblast-like mononuclear cells (Fig. 1c).

The majority of cells covering the bone surface were osteoblasts, staining positive ALP, however little to no osteoclasts stained positive TRAP-5B, cathepsin K or MMP-9 (Fig. 1d).

## 4 Clinical course

Following doxycycline treatment at 100 mg/day, the patient's bone pain improved, and both bone ALP and TRAP-5B levels in the bone declined (Table 2). In addition, skin lesion also subsided.

**Table 2:** Bone marker before and after treatment. ALP, alkaline phosphatase; iPTH, intact parathyroid hormone.

	Before treatment	After treatment	Normal range	
Bone specific ALP	90.8	22.8	3.8–22.6	μg/L
TRACP-5b	965	514	250–760	mU/dL
1,25dihydroxyvitaminD	54	26	20.0–60.0	ng/L
Corrected Ca	9	9.5	8.7–10.1	mg/dL
iPTH	177	39	15–65	pg/mL
Osteocalcin	26.7	26.1	14.2–54.8	ng/mL
Deoxypyridinoline(Urine)	12.2	23	2.8–7.6	nmol/mmol･Cr

## 5 Discussion

The criteria formulated by Kahn et al. are widely used for the diagnosis of SAPHO syndrome (Rukavina, 2015; Hayem, 2004). According to this criteria, SAPHO syndrome can be diagnosed by: bone-joint pain associated with palmoplantar pustulosis, psoriasis vulgaris, severe acne or chronic bowel diseases; isolated sterile hyperostosis/osteitis, and chronic recurrent multifocal osteomyelitis. Using this criteria, several cases have been reported in which SAPHO syndrome has been diagnosed with only hyperostosis and not synovitis (Collange et al., 1996; Cotten et al., 1995; Orui et al., 2002). In previous reports (Collange et al., 1996; Cotten et al., 1995; Orui et al., 2002), the long bone lesions of SAPHO syndrome have occurred in approximately 30% of the patients, including osteosclerosis, osteolysis and hyperostosis. It is well known that hyperostosis is involved in the shaft of long bones. The most common site of hyperostosis is the distal femur and proximal tibia, however, the fibula, humerus, radius, and ulna can also be affected (Cotten et al., 1995; Orui et al., 2002). Hyperostosis is, however, rarely involved in epiphyseal lesion.

The pathogenesis of localized hyperostosis currently remains unknown. A case study by Mokuda et al. reported sternocostoclavicular hyperostosis (SCCH) following bone histomorphometrical analysis of a 77-year-old Japanese man suffering from SAPHO syndrome. The study showed that in normal bone, cancellous bone showed double labeling indicating normal turnover, and cortical bone showed no labeling indicating low turnover or an adynamic state. In the patient, however, cancellous bone showed no labeling and cortical bone displayed double labeling. It was therefore concluded that this abundant cortical bone formation may contribute to sternocostoclavicular hyperostosis (Mokuda et al., 2010).

The antibiotic therapy including azithromycin, clindamycin and doxycycline has been reported to be effective for patients with SAPHO syndrome (Assmann et al., 2009). Therefore doxycycline was selected on this case.

The pathogenesis of the SAPHO spectrum remains unknown but there are also hypotheses of infectious disease, suggesting that bone lesions are caused by a low-virulence pathogen such as *Propionibacterium acnes* (Rukavina, 2015). Our patient had severe focal skin lesion on feet and fingers, and palmoplantar pustulosis was diagnosed. Our patient's bone pain and skin lesion subsided following doxycycline treatment. This indicates that any pathogen originated from these skin lesion might land on the outer portion of the femur, and might have contributed to the formation of the bone lesion, although definite pathogen was not cultured.

In conclusion, histomorphometrical analysis of a patient suffering from SAPHO syndrome showed that both cancellous and cortical bone showed abundant osteoid and woven bone with markedly double labeling (indicating increased turn over). In addition, immunohistology revealed that the majority of the cell lines covering the bone surface consisted of osteoblasts, staining positive for ALP. The findings of the current study indicate excessive production of osteoblasts, but not osteoclasts, contributes to increased bone formation, including abundant osteoid and woven bone, in the SAPHO syndrome. This type of bone formation in SAPHO syndrome is not lamellar bone seen in normal bone, but rather fragile and mechanically weak bone, resulting in bone pain. Unfortunately, the authors were unable to determine why hyperostosis was restricted to only the left femur in this case study. Further investigations are required with a larger sample size to provide a clearer understanding of this particular type of hyperostosis.

## Transparency document

Transparency document. Image 1

## Declaration of competing interest

Shun Watanabe, Naoki Sawa, Hiroki Mizuno, Rikako Hiramatsu, Noriko Hayami, Masayuki Yamanouchi, Tatsuya Suwabe, Junichi Hoshino, Takeshi Fujii, Toshihide Hirai, Tomoka Hasegawa, Norio Amizuka, and Yoshifumi Ubara declare that they have no conflict of interest.
